# Factors affecting local control of bone metastases from radioresistant tumors treated with palliative external beam radiotherapy

**DOI:** 10.1007/s12672-023-00651-0

**Published:** 2023-05-22

**Authors:** Kenji Makita, Yasushi Hamamoto, Hiromitsu Kanzaki, Kei Nagasaki, Noriko Takata, Shintaro Tsuruoka, Kotaro Uwatsu, Teruhito Kido

**Affiliations:** 1 Department of Radiology, Ehime University Graduate School of Medicine, 454 Shitsukawa, Toon, Ehime 791-0295 Japan; 2 Departments of Radiation Oncology, National Hospital Organization Shikoku Cancer Center, Kou-160, Minami-Umenomoto-Machi, Matsuyama, Ehime 791-0280 Japan

**Keywords:** Bone metastases, Local control, Radioresistant carcinoma, External-beam radiotherapy, Dose escalation

## Abstract

**Background:**

This study aimed to evaluate the factors that affect the local control (LC) of bone metastases from radioresistant carcinomas (renal cell carcinoma, hepatocellular carcinoma [HCC], and colorectal carcinoma [CRC]) treated with palliative external-beam radiotherapy (EBRT).

**Methods and materials:**

Between January 2010 and December 2020, 211 bone metastases in 134 patients were treated with EBRT in two hospitals (a cancer center and university hospital). Based on follow-up CT, these cases were reviewed retrospectively to evaluate LC at the EBRT site.

**Results:**

The median EBRT dose (BED10) was 39.0 Gy (range, 14.4–66.3 Gy). The median follow-up time of the imaging studies was 6 months (range, 1–107 months). The 0.5-year overall survival and LC rates of the EBRT sites were 73% and 73%, respectively. Multivariate analysis revealed that the primary sites (HCC/CRC), low EBRT dose (BED10) (≤ 39.0 Gy), and non-administration of post-EBRT bone modifying agents (BMAs) and/or antineoplastic agents (ATs) were statistically significant factors that negatively affected the LC of EBRT sites. In the absence of BMAs or ATs, the EBRT dose (BED10) escalation from 39.0 Gy improved the LC of EBRT sites. Based on ATs administration, the LC of EBRT sites was significantly affected by tyrosine kinase inhibitors and/or immune checkpoint inhibitors.

**Conclusions:**

Dose escalation improves LC in bone metastases from radioresistant carcinomas. Higher EBRT doses are needed to treat patients for whom few effective systemic therapies remain available.

## Background

At the time of death, 70–85% of patients with advanced carcinoma have bone metastases [1]. The incidence of bone metastases varies depending on the primary site and is approximately 30% in patients with renal cell carcinoma (RCC), 15% in patients with hepatocellular carcinoma (HCC), and 10% in patients with colorectal carcinoma (CRC) [2–4].

Bone metastases are associated with symptoms such as hypercalcemia and skeletal-related events, such as a pathologic fracture, spinal cord compression, the necessity for radiation to bone (for pain or impending fracture), or bone surgery [5]. External-beam radiotherapy (EBRT) is a common treatment for bone metastases. Many guidelines for managing bone metastases have recommended single-fraction EBRT at 8 Gy for painful bone metastases in recent years [6–8]. However, the optimal palliative EBRT dose for painful bone metastases caused by radioresistant carcinomas remains unclear [9, 10]. Furthermore, in a recent nationwide study, only < 10% of palliative courses used single-fraction EBRT at 8 Gy for bone metastases [11]. One possible explanation is that many radiation oncologists select fractionated EBRT when some degree of local control (LC) of EBRT sites is desired in patients with bone metastases with a predicted comparatively long-term prognosis. Because higher EBRT doses are required for LC of radioresistant carcinomas bone metastases compared to radiosensitive carcinomas bone metastases, a single-fraction EBRT at 8 Gy could not be uniformly recommended in patients with a predicted comparatively long-term prognosis.

Recently, because significant progress in systemic therapies has improved the expected prognoses of patients with the three (RCC, HCC, and CRC) advanced carcinomas mentioned above [12–14], the number of patients with long-term prognoses who have multiple bone metastases is likely to increase. Therefore, local control (LC) of bone metastases in these radioresistant carcinomas will become more important in the future because local enlargement of bone metastases has the potential to cause neurological symptoms in addition to pain increase. Nevertheless, except for metastatic spinal cord compression, few studies have investigated LC using EBRT for bone metastases [15, 16], and LC using EBRT for bone metastases from these radioresistant carcinomas has not been well documented. Therefore, this study aimed to investigate the LC of all bone metastases from radioresistant carcinomas after palliative EBRT.

## Methods

In this study, the LC of radioresistant carcinomas, including RCC, HCC, and CRC, was examined. These radioresistant carcinomas were classified based on previous studies [17, 18]. The Ethics Committee of Ehime University Hospital (registration number: 1912010) and National Hospital Organization Shikoku Cancer Center approved this retrospective study (registration number: RIN2019-79).

LC was evaluated using non-contact computed tomography (CT) on an irregular basis. Local failure was defined as the enlargement of the lytic change or extraosseous mass at the EBRT sites compared to the size of the lesions before EBRT. Similarly, bone metastases at non-EBRT sites were assessed for the enlargement of lytic changes or extraosseous masses compared to the size of lesions at the time of palliative EBRT. The CT images evaluated for LC at EBRT and non-EBRT sites were taken at the same data and time. During image evaluation, two observers (a radiologist and radiation oncologists) were blinded to the follow-up information and outcomes. Further, when the evaluations differed between the two observers, they discussed the results and reached a consensus.

Three-dimensional conformal radiation therapy was used for all EBRTs. EBRT was administered using 6–10 MV X-rays from linear accelerators (Clinac iX, Clinac 21EX, or TrueBeam; Varian Medical Systems, CA, USA). In principle, the target volume dose was prescribed to be ≥ 90% of the EBRT dose. The doses were calculated using the anisotropic analytical algorithm of our treatment planning systems (Eclipse planning system: Varian Medical Systems, CA, USA). In addition, the biologically effective dose (BED) was calculated to compare the various fractionated schedules. The BED10 (BED calculated using an α/β of 10 Gy) was calculated with nd (1 + d/(α/β)), where d is the fraction dose, n is the number of fractions, and α/β is 10 Gy.

Each attending physician had complete discretion over administering bone-modifying agents (BMAs) and antineoplastic agents (ATs). In addition, pre-EBRT neutrophil–lymphocyte (NLR) and platelet-to-lymphocyte (PLR) ratios were assessed as host inflammatory response markers, which correlated with carcinomas aggressiveness in several carcinomas [19, 20].

The time of survival and LC of the EBRT sites were calculated from the start of palliative EBRT. The Kaplan–Meier method was used to generate overall survival (OS) and LC curves. We used univariate and multivariate Cox proportional hazards models to determine hazard ratios (HRs), including 95% confidence intervals (CIs) and p-values. Variables included in the multivariate models had a p-value < 0.05 in the univariate analysis. Statistical significance was defined as a p-value < 0.05. Statistical analyses were performed using the JMP software (JMP version 14.3.0; SAS Institute, Cary, NC, USA).

## Results

Between January 2010 and December 2020, palliative EBRT was used to treat 331 metastatic bone lesions in 216 patients at the National Hospital Organization Shikoku Cancer Center (n = 180) and Ehime University Hospital (n = 151). The exclusion criteria were as follows from the above: (1) pathologic fracture without surgical treatment (n = 4); (2) surgical treatment (n = 8); (3) non-massive bone metastases (n = 9); (4) follow-up time of < a month excluding regrowth (n = 11); and (5) absence of follow-up computed tomography (CT) data (n = 86). Finally, 134 patients (male/female = 96/39; age, median [range]: 66 [36–88] years, HCC/RCC/CRC = 50/49/35) with 211 bone metastatic lesions were followed up with CT after EBRT treatment. This retrospective analysis study was used to evaluate the LC of EBRT and non-EBRT sites in these patients.

The EBRT doses were determined at the discretion of each physician and institution. The median BED10 of EBRT was 39.0 Gy (range, 14.4–66.3 Gy). The following dose fraction schedules [BED10] were used; 1 × 8 Gy [14.4 Gy] (n = 8), 5 × 4 Gy [28.0 Gy] (n = 24), 6 × 4 Gy [33.6 Gy] (n = 1), 8 × 3.6 Gy [29.4 Gy] (n = 1), 10 × 3 Gy [39.0 Gy] (n = 113), 12 × 3 Gy [46.8 Gy] (n = 3), 13 × 3 Gy [50.7 Gy] (n = 7), 15 × 3 Gy [58.5 Gy] (n = 2), 17 × 3 Gy [66.3 Gy] (n = 1), 14 × 2.5 Gy [43.8 Gy] (n = 2), 15 × 2.5 Gy [46.9 Gy] (n = 4), 16 × 2.5 Gy [50.0 Gy] (n = 37), 25 × 2 [60.0 Gy] (n = 5), 14 × 1.8 Gy [29.7 Gy] (n = 1), and 5 × 4 Gy + 3 × 3 Gy [39.7 Gy] (n = 2).

The median and radiographic follow-up times were 9 months (range, 1–140 months) and 6 months (range, 1–107 months), respectively. There were 107 vertebral, 51 pelvic, 23 rib, and 30 other bone metastases cases. Table 1 lists the details of these characteristics.

**Table 1:** Characteristics of lesions

Factors		EBRT (BED10) ≤ 39.0 Gy arm	EBRT (BED10) > 39.0 Gy arm
Age, n (%)	< 65 years	64 (43.2)	39 (61.9)
	≥ 65 years	84 (56.8)	24 (38.1)
Sex, n (%)	Male	96 (64.9)	50 (79.4)
	Female	52 (35.1)	13 (20.6)
ECOG-PS, n (%)	< 2	68 (45.9)	32 (50.8)
	≥ 2	74 (50.0)	23 (36.5)
	Unknown	6 (4.1)	8 (12.7)
Primary sites, n (%)	Hepatocellular carcinoma	66 (44.6)	22 (34.9)
	Renal cell carcinoma	30 (20.3)	9 (14.3)
	Colorectal carcinoma	52 (35.1)	32 (50.8)
Metastases on internal organs, n (%)	Yes	123 (83.1)	45 (71.4)
	No	25 (16.9)	18 (28.6)
Number of bone metastatic lesions, n (%)	Single	31 (21.0)	18 (28.6)
	≤ 3	13 (8.8)	8 (12.7)
	> 3	104 (70.3)	37 (58.7)
EBRT sites, n (%)	Vertebral	78 (52.7)	29 (46.0)
	Pelvis	38 (25.7)	13 (20.6)
	Others	32 (21.6)	21 (33.3)
Bone cortex destruction, n (%)	Yes	44 (29.7)	19 (30.2)
	No	104 (70.3)	44 (69.8)
Post-EBRT BMAs, n (%)	Yes	84 (56.8)	43 (68.3)
	No	64 (43.2)	20 (31.8)
Pre-EBRT ATs, n (%)	Yes	79 (53.4)	27 (42.9)
	No	69 (46.6)	36 (57.1)
Post-EBRT ATs, n (%)	Yes	107 (72.3)	49 (77.8)
	Tyrosine kinase inhibitors	54 (25.6)	30 (47.6)
	Immune checkpoint inhibitors	12 (5.7)	14 (22.2)
	No	41 (27.7)	14 (22.2)
Pre-EBRT laboratory data, n (%)	NLR		
	≤ 3.58	67 (45.3)	39 (61.9)
	> 3.58	81 (54.7)	24 (38.1)
	PLR		
	≤ 6.50	77 (52.0)	31 (49.2)
	> 6.50	71 (48.0)	32 (50.8)

ECOG PS, Eastern Cooperative Oncology Group Performance Status; EBRT, external beam radiotherapy; BED, biologically effective dose; BMAs, bone modifying agents; ATs, antineoplastic agents; NLR, neutrophil to lymphocyte ratio; PLR, platelet to lymphocyte ratio

**Fig. 1:**
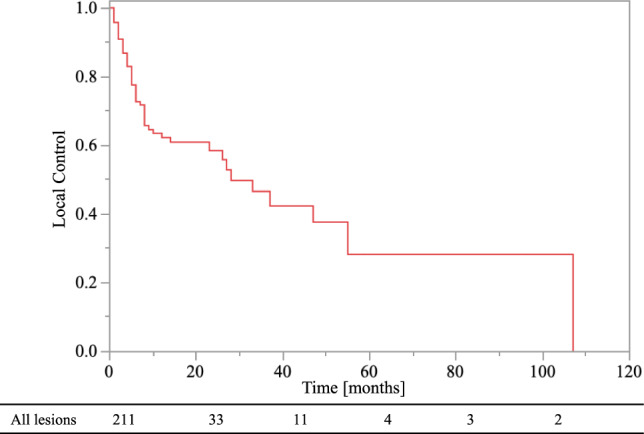
Local control of all bone metastatic lesions

The 0.5- and 1-year OS rates were 73% and 48%, respectively. The 0.5- and 1-year LC rates of the EBRT sites were 73% and 62%, respectively (Fig. 1). Local recurrence at EBRT sites was observed in 30.3% (n = 64) of lesions, and the median time to recurrence was 5 months (range, 1–107 months). The average survival time following local enlargement was 2 months (range, 0–33 months). In addition, the 0.5- and 1-year control rates were 56% and 38% for non-EBRT bone metastatic sites, respectively. At the time of local recurrence or the last CT evaluation of the EBRT sites, 61.7% (n = 129) of the lesions had local enlargement of non-EBRT bone metastatic sites. Further, even though the EBRT site was controlled, 69.0% (n = 89) of these patients developed non-EBRT bone metastatic sites. In contrast, local control of EBRT sites was achieved in 69.7% of cases (n = 147), and 39.5% (n = 58) of these had local control over non-EBRT sites.

### Influence of treatment-related risk factors

Treatment-related risk factors included the EBRT dose (BED10), administration of BMAs, administration of ATs before EBRT (pre-EBRT ATs), and administration of ATs after EBRT (post-EBRT ATs).

#### Radiotherapy

**Fig. 2:**
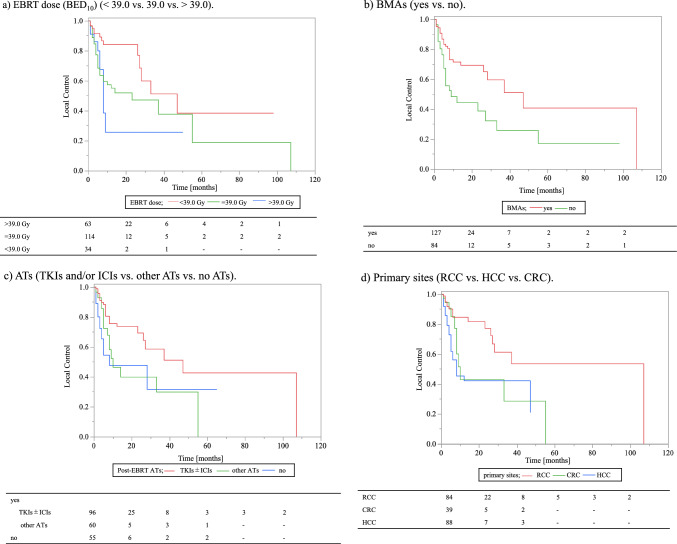
Local control of bone metastases. **a** EBRT dose (BED_10_) (< 39.0 vs. 39.0 vs. > 39.0). **b** BMAs (yes vs. no). **c** ATs (TKIs and/or ICIs vs. other ATs vs. no ATs). **d** Primary sites (RCC vs. HCC vs. CRC)

The 0.5-year LC rates of EBRT sites based on EBRT dose (BED10) significantly differed between cases with EBRT dose (BED10) = 39.0 Gy and EBRT dose (BED10) > 39.0 Gy (64% vs. 89%; HR, 0.43; 95% CI 0.23–0.80; p = 0.01, Fig. 2a, Table 2). In contrast, there were no significant differences between EBRT dose (BED10) = 39.0 Gy and EBRT dose (BED10) < 39.0 Gy (64% vs. 68%; HR 1.24; 95% CI 0.63–2.43; p = 0.53, Fig. 2, Table 2).

**Table 2:** Local control rates after EBRT and results of univariate and multivariate analyses

		0.5-year (%)	1-year (%)	Univariate analysis		Multivariate analysis	
				HR (95% CI)	*P*	HR (95% CI)	*P*
Age	< 65 years vs. ≥ 65 years	77 vs. 69	63 vs. 62	1.40 (0.84–2.33)	0.19	–	–
Sex	Female vs. male	74 vs. 72	56 vs. 65	1.47 (0.86–2.51)	0.16	–	–
Primary sites	RCC vs. HCC + CRC	85 vs. 64	85 vs. 43	3.10 (1.73–5.53)	< 0.01	2.39 (1.30–4.40)	0.01
Metastases on internal organs	Yes vs. no	73 vs. 71	59 vs. 68	0.78 (0.43–1.43)	0.43	–	–
Number of bone metastatic lesions	< 4 vs. ≥ 4	70 vs. 74	58 vs. 64	1.21 (0.73–2.04)	0.45	–	–
EBRTsites	Vertebral vs. others	71 vs. 74	66 vs. 59	1.15 (0.70–1.89)	0.58	–	–
Bone cortex destruction	Yes vs. no	67 vs. 86	62 vs. 63	1.38 (0.79–2.41)	0.26	–	–
EBRT dose (BED10)	39.0 Gy vs. < 39.0 Gy	64 vs. 68	55 vs. 26	1.24 (0.63–2.43)	0.53	1.29 (0.66–2.55)	0.46
	39.0 Gy vs. > 39.0 Gy	64 vs. 89	55 vs. 84	0.43 (0.23–0.80)	0.01	0.46 (0.24–0.88)	0.02
Post-EBRT BMAs	Yes vs. no	82 vs. 56	72 vs. 44	2.13 (1.29–3.51)	< 0.01	1.94 (1.17–3.23)	0.01
Pre-EBRT ATs	Yes vs. no	70 vs. 75	58 vs. 66	0.79 (0.48–1.30)	0.35	–	–
Post-EBRT ATs	Yes vs. no	78 vs. 55	66 vs. 48	2.20 (1.27–3.80)	< 0.01	2.19 (1.23–3.92)	0.01
Pre-EBRT NLR	< 3.58 vs. ≥ 3.58	74 vs. 71	66 vs. 55	1.08 (0.65–1.79)	0.77	–	–
Pre-BERT PLR	< 6.50 vs. ≥ 6.50	74 vs. 72	58 vs. 66	0.76 (0.45–1.26)	0.28	–	–

RCC, enal cell carcinoma; HCC, hepatocellular carcinoma; CRC, colorectal carcinoma; EBRT, external beam radiotherapy; BED, biologically effective dose; BMAs, bone modified agents; ATs, antineoplastic agents; NLR, neutrophil to lymphocyte ratio; PLR, platelet to lymphocyte ratio

#### Bone modifying agent

The 0.5-year LC rates of EBRT sites based on post-EBRT BMAs significantly differed between cases with and without post-EBRT BMAs (82% vs. 56%; HR, 2.13; 95% CI 1.29–3.51; p < 0.01, Fig. 2b, Table 2).

In cases with post-EBRT BMAs, there were no statistically significant differences between cases with an EBRT dose (BED10) ≤ 39.0 Gy and EBRT dose (BED10) > 39.0 Gy (HR, 0.72; 95% CI 0.34–1.55; p = 0.40). However, in cases without post-EBRT BMAs, there were statistically significant differences between cases with an EBRT dose (BED10) ≤ 39.0 Gy and EBRT dose (BED10) > 3 9.0 Gy (HR, 0.16; 95%CI 0.05–0.54; p < 0.01).

#### Antineoplastic agent

The 0.5-year LC rates of EBRT sites based on post-EBRT ATs were significantly different between cases with and without post-EBRT ATs (78% vs. 55%; HR, 2.20; 95% CI 1.27–3.80; p < 0.01, Fig. 2c, Table 2). Sixty-two patients with 99 lesions had ATs at the time of local recurrence or the last CT evaluation of the EBRT sites, while the other 72 patients with 112 lesions did.

In cases with post-EBRT ATs, there were no statistically significant differences between EBRT dose (BED10) ≤ 39.0 Gy and EBRT dose (BED10) > 39.0 Gy (HR, 0.54; 95%CI 0.27–1.07; p = 0.08). However, in cases without post-EBRT ATs, there were statistically significant differences between EBRT dose (BED10) ≤ 39.0 Gy and EBRT dose (BED10) > 39.0 Gy (HR, 0.09; 95%CI 0.01–0.73; p = 0.02).

Post-EBRT ATs were (1) tyrosine kinase inhibitors (TKIs) [n = 65], (2) immune checkpoint inhibitors (ICIs) [n = 7], (3) TKIs + ICIs [n = 19], and (4) others [n = 60]. Post-EBRT ATs were divided into two groups: TKIs and/or ICIs [n = 96] and others [n = 60], according to the 0.5-year LC rates (TKIs, 78.2%; ICIs, 85.7%; TKIs + ICIs, 88.2%; other, 53.1%). There were statistically significant differences between the TKIs and/or ICIs group and the others (0.5-year LC rates, 81.8% vs. 53.1%; HR, 2.05; 95% CI 1.12–3.75; p = 0.02).

### Influence of carcinoma-related risk factors

As carcinoma-related risk factors, primary sites, metastases to internal organs, the number of bone metastatic lesions, EBRT sites, and bone cortex destruction were evaluated.

The 0.5-year LC rates of EBRT sites based on primary sites differed significantly between RCC and HCC (85% vs. 56%; HR, 3.58; 95% CI 1.95–6.58; p < 0.01) and between RCC and CRC (85% vs. 85%; HR, 2.21; 95% CI 1.02–4.78; p = 0.05, Fig. 2d). In contrast, there were no significant differences in the LC of the EBRT sites between HCC and CRC (HR, 0.62; 95% CI 0.31–1.22; p = 0.16). In addition, there were no statistically significant differences in the LC of the EBRT sites between vertebral and other bone metastatic sites (Table 2).

### Influence of patient-related risk factors

Age, sex, NLR, and PLR were analyzed as patient-related risk factors. The median pre-EBRT NLR and PLR were 3.58 (range, 0.89–34.69) and 6.50 (range, 0.49–74.75), respectively.

None of these variables differed significantly between the LC and EBRT sites (Table 2). In 197 lesions, excluding those with unknown performance status (PS), the 0.5-year LC rates of EBRT sites were not significantly different between cases with a PS < 2 and those with a PS ≥ 2 (72% vs. 72%; HR, 1.15; 95% CI, 0.69–1.90; p = 0.60).

### Multivariate Cox regression analysis

HCC/CRC, EBRT dose (BED10) ≤ 39.0 Gy, no post-EBRT BMAs, and no post-EBRT ATs were statistically significant unfavorable factors for the LC of bone metastases in the multivariate analysis (Table 2).

## Discussion

In this study, the 1-year OS rate in patients who required palliative EBRT for bone metastases from radioresistant carcinomas was approximately 50%. The primary sites, EBRT dose (BED10), and post-EBRT BMA/ATs played an important role in the LC of bone metastases following palliative EBRT. In cases without BMAs or ATs, the EBRT dose (BED10) escalation from 39.0 Gy significantly improved the LC of bone metastases. TKIs and/or ICIs have a comparatively significant impact on the LC of EBRT sites regarding ATs.

EBRT reduced local failure of EBRT sites, even when conventional palliative EBRT doses were used. Previous studies have revealed that the moderate EBRT dose (BED10) escalation from 39.0 Gy had a minimal impact on improving the LC of EBRT sites [17, 21]. However, EBRT dose escalation for radioresistant carcinomas was effective in this study. According to some studies, higher EBRT doses are important for LC and pain relief in patients with radioresistant carcinomas [9, 22]. In patients with favorable prognoses, radioresistant carcinomas may necessitate a higher EBRT dose.

Previous studies have suggested that primary sites are important for LC at EBRT sites, and radioresistant carcinomas have been divided into two groups (moderately unfavorable, RCC; unfavorable, HCC, and CRC) [17, 21]. In this study, LC at EBRT sites differed according to the primary sites, with HCC/CRC bone metastases showing more unfavorable LC than those from RCC. Stereotactic body radiotherapy (SBRT) is a sophisticated technique for administering extremely high doses of radiation to bone metastases. Compared to conventional EBRT, SBRT achieved better LC and pain control and lower re-irradiation rates [23, 24]. In RCC and HCC, SBRT has been shown to provide adequate LC of bone metastases [25–27]. Therefore, in patients with a favorable prognosis, these radioresistant carcinomas may respond better to SBRT than conventional palliative EBRT. In contrast, while SBRT for bone metastasis from CRC would provide significant improvement compared to conventional palliative EBRT, it does not achieve adequate LC of irradiated sites [28]. In our study, post-EBRT ATs and BMAs improved the LC of EBRT sites. Similarly, some studies have suggested that ATs and BMAs improve the LC of bone metastases [29, 30]. Furthermore, combining radiotherapy with ATs and/or BMAs can be beneficial [31–33]. The use of BMAs and/or ATs combined with EBRT is recommended for bone metastases from radioresistant carcinomas; especially, bone metastases from CRC warrant the addition of BMAs and/or ATs because achieving favorable LC in these cases is extremely challenging.

In this study, although the impact of EBRT dose escalation (BED10) from 39.0 Gy on LC was strong in patients who were not administered ATs or BMAs following EBRT, the impact on LC was minimal in patients who were administered ATs or BMAs after EBRT. Furthermore, TKI and/or ICI administration significantly affected the LC of the EBRT sites. Some studies have suggested that treating patients with bone metastases using TKIs and/or ICIs, and BMAs is beneficial [34, 35]. Therefore, when ATs (especially TKIs and/or ICIs) or BMAs could not be used, dose escalation using precise EBRT, such as SBRT [25–28], seemed useful for treating bone metastases in patients with favorable prognoses.

This study had some limitations due to its retrospective nature. First, the number of CRC cases was small compared with that of RCC and HCC. Second, due to the two-institutional and long-term study design, there may have been selection bias in determining EBRT doses, ATs, and BMAs because many radiation oncologists and attending physicians were involved in patient management. Finally, this study did not assess pain relief, skeletal-related events, and various adverse events, all of which are important factors in palliative intent EBRT. In addition, the course of these symptoms after palliative intent EBRT was also challenging to evaluate precisely in a retrospective nature. However, as systemic therapy advances, precision medicine in palliative EBRT is becoming more important, and the LC of bone metastases is becoming increasingly important for patients with favorable prognoses. This study provides a perspective on individualized medicine. Following significant advances in systemic and supportive therapies, the LC rates of bone metastases should be updated regularly in the future.

## Conclusions

Our findings suggested that the EBRT dose escalation from 39.0 Gy (10 × 3 Gy) improved the LC of bone metastases from radioresistant carcinomas. Furthermore, post-EBRT ATs (especially TKIs and/or ICIs) and BMAs had a significant positive impact on the LC of bone metastases, and the impact of EBRT dose escalation (BED10) from 39.0 Gy was minimal when these systemic therapies were used to treat radioresistant carcinomas.

## Data Availability

Not applicable.
